# Clinico-microbiological investigation of factors contributing to suboptimal viral suppression among HIV/AIDS patients on sustained antiretroviral treatment in the upper east region of Ghana

**DOI:** 10.3389/fmed.2026.1837206

**Published:** 2026-09-09

**Authors:** Ebenezer Yeboah, Lawrence Adetunde, Samuel Achagepungu Nikaane, Richmond Balinia Adda, Eugene Dogkotenge Kuugbee

**Affiliations:** 1 Department of Applied Biology, University of Technology and Applied Sciences, Navrongo, Ghana; 2 Department of Medical Laboratory Sciences, College of Health, Kintampo, Ghana; 3 Department of Biostatistics and Epidemiology, School of Public Health, University of Technology and Applied Sciences Ghana, Navrongo, Ghana; 4 Department of Medical Laboratory Science, School of Medicine, University of Technology and Applied Sciences, Navrongo, Ghana

**Keywords:** adherence, antiretroviral therapy, HIV, target not detected, TND, upper east region, viral load suppression

## Abstract

**Introduction:**

HIV remains a major global health challenge, with approximately 48.1 million people affected worldwide and nearly 69% residing in sub-Saharan Africa. Regional disparities remain in Ghana, especially in the Upper East Region, which continues to lag behind the national target rates for viral suppression, despite advances made in expanding access to antiretroviral therapy (ART). This study aimed to examine the clinical factors associated with sub-optimal viral suppression to guide efforts to meet the UNAIDS 95 95 95 targets.

**Methods:**

A cross sectional survey was carried out with 281 HIV positive persons on sustained ART in three major health institutions. Structured questionnaires, clinical record review, and laboratory tests such as hematology, liver function, renal function, metabolic syndrome, and microbiological testing were used for data collection. Viral load results were stratified into four categories: Target Not Detected (TND, <20 copies/mL), optimal suppression (20–199 copies/mL), suboptimal suppression (200–999 copies/mL), and virological failure (≥1,000 copies/mL). Chi-square tests and logistic regression were used for statistical analyses to identify associated factors.

**Results:**

The findings showed that 69.4% of the participants achieved TND, 7.8% had optimal suppression, 17.8% obtained suboptimal suppression, and 5.0% had virological failure. Suppression outcomes were significantly different for ART regimen (*p* = 0.001): patients receiving AZT/3TC/NVP were more likely to achieve TND than those receiving TLD (AOR = 1.42, 95% CI: 1.11–2.34). Duration on ART also influenced suppression (*p* = 0.03): people who were on ART for 11–15 years were more likely to be well-suppressed (AOR = 1.56, 95% CI: 1.08–2.25) than those on ART for 1–5 years. Oddly enough, defaulters had paradoxically higher odds of TND (AOR = 1.31, *p* = 0.036). Biochemical markers such as total protein (AOR = 1.47, *p* = 0.042) and albumin/globulin ratio (AOR = 1.52, *p* = 0.029) were found to be significantly associated with suppression. Systemic infections (bacteremia and urinary tract infections) had a tendency toward worse outcomes but the relationship was not statistically significant.

**Conclusion:**

ART regimen, treatment duration, ART adherence history and biochemical markers were found to be important factors of viral suppression in the Upper East Region.

## 1 Introduction

Human Immunodeficiency Virus (HIV) is one of the most important worldwide issues of social health, despite biomedical breakthroughs. As of the end of 2024, it was estimated that 40.8 million people were living with HIV. It continues to be disproportionately affected by sub-Saharan countries (1). Despite the current improvement in treatment with antiretroviral therapy (ART), HIV infection remains an important public health problem, particularly in sub-Saharan Africa, where the burden of the disease is the highest (1). HIV treatment has transformed with the advent of highly active antiretroviral therapy (HAART), which allows for effective viral suppression, leading to reduced morbidity, mortality, and HIV transmission (2). The main objectives of HIV treatment include viral suppression, restoring immune system function, and decreasing HIV transmission. Treatment is effective when a person’s HIV viral load is below 1,000 copies per milligram of blood, and the best outcomes from treatment occur when the HIV viral load falls below 20 copies per milligram of blood or undetectable (3). However, the proportion of patients not virally suppressed and of those with virological failure remains significant, especially in resource-limited settings, among patients on sustained ART (4). Ghana has experienced successful efforts to achieve the UNAIDS goals of 95–95–95, leading to improved access to HIV diagnosis and treatment, but with some variation between regions (5). A study conducted in the Upper East Region revealed that although viral suppression continues to lag national and global targets, a high percentage of individuals on ART in the region were not fully suppressed (6). This is a major clinical and public health problem in the region, as it implies the development of drug-resistant viral strains, failure of treatment, HIV transmission, and disease progression (7). Co-infection—especially gastrointestinal infections, blood stream infections and urinary tract infections (UTIs)—are common in resource-limited areas and may be associated with poor treatment responses and chronic immune activation and inflammation (8). HIV infection, ART toxicity, and concurrent infections are also the main causes of hematological abnormalities, including anemia, leukopenia, thrombocytopenia, and abnormalities in red blood cell morphology commonly observed among the HIV cases (9). Moreover, several ART-related toxicities like hepatotoxicity, nephrotoxicity, dyslipidemia, and metabolic disorders could impact treatment adherence and therapeutic success (10). Structural and socio-demographic issues further complicate treatment of HIV in northern Ghana (6). Poor ART adherence, distance to healthcare services, food insecurity, stigma, and discontinuity in the provision of ART may be factors affecting viral suppression in people on long-term ART (11). Furthermore, in many resource-limited settings, there is also limited laboratory capacity for routine laboratory investigations or patient monitoring, or to detect and manage complications related to treatment (12). While few studies in Ghana have investigated adherence to antiretroviral therapy (ART) and the general predictors of virological failure, few have comprehensively examined the combined influence of systemic infections, hematological abnormalities, ART-related toxicities, and structural factors on viral suppression outcomes, particularly in the Upper East Region. Integrated clinico-microbiological evidence is an important research gap that challenges the development of targeted interventions for patients at high risk of treatment failure. Thus, this study set out to carry out a detailed clinico-microbiological investigation of variables that are linked to suboptimal viral suppression among HIV/AIDS clients on sustained ART in selected health facilities in the Upper East Region of Ghana. The findings from this research will provide evidence for use in the specific context to support improved patient monitoring, more effective HIV treatment program and interventions to improve the results of viral suppression for the region.

## 2 Materials and methods

### 2.1 Study setting

This study was conducted in three purposively selected health institutions, namely, War Memorial Hospital, Navrongo; Sandema District Hospital; and Paga District Hospital in the Upper East Region of Ghana. War Memorial Hospital is situated at Navrongo (10.895°N, 1.093°W) with an altitude of about 201 m above sea level. Sandema District Hospital is located at 10.5290° N, 1.0610° W, with an altitude of about 180 m above sea level, while Paga District Hospital is at 10.9960° N, 1.1070° W, in proximity to the border of Ghana and Burkina Faso, with an altitude of approximately 220 m above sea level. These facilities are key antiretroviral therapy (ART) facilities in two strategic areas (Kasena-Nankana and Builsa North) with a large concentration of people living with HIV/AIDS in the region. The facilities reflected the diversity of urban and rural communities and how they function, as well as the dynamics of border communities. War Memorial Hospitals serve a heterogeneous population, which makes it a suitable hospital to look at various clinical presentations. Sandema District Hospital is a representative example of socio-economic constraints and barriers to access to health care in rural and remote areas. Paga District Hospital is a typical example of a border province with high mobility. Even though the main regional referral hospital was Bolgatanga Regional Hospital, we did not include it in the study because the study aimed to focus on district-level hospitals, for which there was sufficient logistic capacity, to enable the sustained recruitment of participants and extended follow-up of data. These three clinics are thus representative of community-based HIV services in resource-poor settings and serve as a generalizable sample of the community for study design purposes.

### 2.2 Study population

The study was conducted among HIV-positive people taking antiretroviral therapy (ART) treatment in selected health facilities of the Kassena-Nankana and Builsa Municipalities in the Upper East Region of Ghana. Adults with a history of ART treatment varying from newly initiated to long-term use of ART were eligible. Having patients with different treatment durations enabled the analysis of treatment outcomes at each time point of ART exposure and helped to identify potential ART duration dependence. Participants were categorized based on the most recent plasma HIV viral load measurement obtained from the last 12 months, following the criteria outlined in the World Health Organization (WHO) and 2024 Ghana National HIV Treatment Guidelines. The viral load results were stratified as Target Not Detected (<20 copies/mL), Optimal Suppression (20–199 copies/mL), Suboptimal Suppression (200–999 copies/mL), and Virological Failure (the viral load was ≥1,000 copies/mL) (13).

### 2.3 .Viral load categorization

We added a four-level classification in addition to the standard two-level categorization scheme (suppressed as less than 1,000 copies/mL and unsuppressed: >1,000 copies/mL) to provide granularity. The definition of virological failure was ≥1,000 copies/mL as agreed by the WHO and Ghana National HIV Treatment Guidelines and was met by the majority of treatment failures and would warrant more attention to adherence and a consideration of regimen change (13). Those with viral loads in the <20 copies/mL category were classified as target not detected (TND), as this was the lowest undetectable viral load, indicating excellent adherence and low potential for transmission. Optimal suppression (20–199 copies/mL) represented patients whose viral load at that time indicated they were virologically suppressed but still had detectable viremia, meaning they were suppressed according to international standards. Patients grouped as ‘suboptimal suppression’ (200–999 copies/mL) are not experiencing a level of suppression expected to be viewed as failure of therapy per the WHO but are at higher risk for resistance development, increased immune activation, and treatment failure. This subtle classification was deliberately used to describe different degrees of viral control and related determinants of HIV-infected individuals on sustained ART in the Upper East Region, Ghana.

### 2.4 Study design

We used a cross-sectional analytical study design exploring a quantitative research approach to determine factors associated with suboptimal viral suppression among HIV/AIDS patients within the Upper East Region of Ghana on sustained ART. Only participants who started ART treatment within at least 12 months before recruitment were included in the study. Socio-demographic data, viral loads, ART adherence, clinical history, comorbidities, and behavioral information that may be a determinant to the viral suppression outcome were collected using structured questionnaires. Further, clinical and laboratory studies were conducted to determine potential factors involved in treatment outcomes. Laboratory testing included a complete blood count, liver function testing, renal function testing, a lipid profile, urinalysis, routine stool examination, and urine and blood cultures to screen for potential co-infections, metabolic disorders, and hematological abnormalities linking the virological suppression patterns. Using data from questionnaires in addition to the clinical and laboratory data allowed for a comprehensive evaluation of HIV/AIDS clients under continued ART with regard to factors associated with maintaining viral suppression.

### 2.5 Determination of sample size and sampling method

The sample size for the study was determined using the Cochran formula using the national estimate of HIV prevalence of 18% (14), a 95% confidence level, and a margin of error of 5%. The initial calculated sample size was found to be 227 cases. This was adjusted for a 10% nonresponse rate, yielding a sample size of 252 respondents. However, to further strengthen statistical power, reduce the effect of incomplete questionnaires, and mitigate potential data loss or missing information from the questionnaires, the final sample size was increased to 281 participants. A stratified proportionate sampling method was employed to gain a sample representativeness of the selected ART clinics (War Memorial, Sandema, and Paga District). Each hospital was included as a stratum, and a certain number of respondents were sampled in each hospital according to the number of patients in the ART registers of that hospital. The numbers were allocated using Bowley’s formula of proportional allocation (15). After proportional allocation following routine clinic visits, systematic sampling was used for the recruitment. The sampling interval at each facility was the (n) th patient and was obtained by dividing the number of eligible patients attending the clinic during the study period by the HIV sample size of the facility.

### 2.6 Eligibility criteria

#### 2.6.1 Inclusion criteria

Our inclusion criteria included HIV-positive adults aged 18 years and above. HIV participants who had been on ART for at least 12 months were also enrolled in the study. Again, only those who had complete medical records and information regarding demographic, clinical, and treatment information were included to ensure accuracy and reliability in the outcome of treatments.

#### 2.6.2 Exclusion criteria

We excluded patients who were below the age of 18 years from the study. Also, participants who did not have complete or missing medical records, clinical data, and vital demographic information were not included. Those who had been on ART for less than 12 months and pregnant women were excluded.

### 2.7 Data collection procedures

#### 2.7.1 Structured questionnaires

We used structured questionnaires to obtain all the information that provides the demographic data of the participants, such as age, sex, marital status, educational level, occupation, distance to the ART clinic, transportation cost, and duration on ART. Clinicians who were familiar with the local languages such as Frafra, Kasena, and Builsa were involved to help administer the questionnaires to ensure there was clarity. The level of previous familiarity and trust between the patient and clinical personnel helped ward off stigma-related issues, foster a comfortable feel, and elicit truthful and honest responses from the patients. Viral loads of the participants were consecutively checked three times to confirm the differences in the results, and the current results were considered for the stratification of the suppression group. Secondly, the clinical history of the participants was thoroughly examined in order to obtain profound data about their health conditions. These included opportunistic infections and comorbidities (blood pressure and diabetes), ART regimens previously used, and compliance with the ART regimen. This comprehensive extraction gave the chance to make a deep analysis of variables associated with the results of the therapeutic interventions.

### 2.8 Laboratory investigations

#### 2.8.1 Sampling

We used a convenience method for our sample collection. Samples of blood, urine, and stool were collected from the study participants and dispensed in different tubes, namely EDTA, gel, fluoride oxalate, and brain heart infusion (BHI) tubes for different tests. Five milliliters (5 mL) of venous blood was aseptically collected from each subject by a trained laboratory scientist using a Vacutainer needle system following standard phlebotomy protocol. Blood samples were collected in an EDTA tube and gently swirled to mix with the anticoagulant, while the gel tube blood was allowed to coagulate. The serum was separated by spinning in a centrifuge at 3000 rpm for 5–10 min. Clean-catch urine was collected using sterile screw-cap urine containers, and the stool was collected in the clean, labeled stool containers with a cork. Each sample was coded uniquely for the study, with its ART code and a collection date and time. Samples for urine and blood culture were stored at 2–8 °C before processing. The remaining samples for hematology and biochemistry collected in the ART clinics were stored in an ice pack and transported to the laboratory within 1 to 2 h of collection for their analysis. Ideal biosafety conditions during the collection procedure and handling were observed.

#### 2.8.2 Quality control procedures

Internal quality control (IQC) and quality assurance (QA) procedures were carefully established to ensure the reliability and repeatability of all laboratory studies. All analyses were carried out using approved standard operating procedures (SOPs) based on the instructions given by the manufacturer and following the internationally recognized standards: ISO 15189 for medical laboratory competence and quality and ISO 15190 for biosafety requirements in laboratories. Only quality control materials of low, normal, and high levels that meet certain specified acceptable ranges were accepted for quality control. Hematological investigations using the Sysmex analyzer were performed and analyzed daily using the daily IQC, background check, and reagent monitoring. Routine calibration and multi-level serum control verification were performed by biochemical analyses using the Mindray analyzer. Culture media were tested for sterility and performance with reference organisms, and antibiotic susceptibility testing was performed according to the Clinical and Laboratory Standards Institute (CLSI) and the European Committee on Antimicrobial Susceptibility Testing (EUCAST) guidelines for microbiology. The samples were all coded individually and carried in a temperature-controlled environment to maintain their integrity. We carried out the investigations under strict biosafety protocols, and senior scientists confirmed the findings of the investigations, which made the study clinically valid and consistent.

#### 2.8.3 Hematological analysis

2 mL of the collected venous blood was immediately transferred into ethylenediaminetetraacetic acid (EDTA) tubes to avoid coagulation and to prevent loss of cell components. All participants were given an individual participant code to enable accurate tracking and confidentiality. Samples that were collected were inverted gently to mix with the anticoagulant and sent to the laboratory under controlled conditions. All hematological investigations were carried out using an automated hematology analyzer (Sysmex XN-350), which was calibrated and used according to the manufacturer’s instructions and laboratory quality control procedures. The analyzer generated complete blood counts (CBCs), including red blood cell (RBC) count, hemoglobin concentration (Hb), hematocrit (HCT), mean corpuscular hemoglobin concentration (MCHC), total white blood cell (WBC) count, platelet count, and differential leukocyte counts.

#### 2.8.4 Biochemical analysis

4 mL of the collected venous blood was dispensed into a gel-separating tube, allowed to clot, and then centrifuged at 3000 rpm for 10 min to separate the serum from the red cells. The Mindray chemistry analyzer (BS-240) was used for biochemical analyses such as liver function tests to measure ALT, ALP, AST, GGT, bilirubin, total protein, and albumin concentrations, as well as renal function tests to measure creatinine, urea, blood urea nitrogen (BUN), and serum electrolytes (Sodium, potassium, and chloride). The Cockcroft formula was used to determine the estimated glomerular filtration rate to determine the strength of the kidneys of the participants. Metabolic markers, such as lipid profile (total cholesterol, triglycerides, high-density lipoprotein (HDL-C), low-density lipoprotein (LDL-C), and glucose concentration), were also measured using the same analyzer. Body Mass Index (BMI) was determined by dividing weight by height (weight in kg/height in m^2^). Other laboratory indices calculated were the AST/ALT ratio and the albumin/globulin ratio (A/G). We further used NCEP ATP III standards to assess the participants on the indicators of metabolic syndrome to offer decentralized judgment of indicative biochemical health conditions.

#### 2.8.5 Microbiological analysis

Detailed microbiological studies were conducted to reveal bacterial and parasitic infections in patients with HIV/AIDS attending ART clinics. Such investigations were urine cultures, blood cultures, and routine examination of stool and urine samples to identify typical pathogens and parasitic infections, which include urinary tract infections (UTIs) and gastrointestinal infections (GIT) such as Giardiasis, intestinal flagellates, Strongyloidiasis, amoebiasis, and cryptosporidiosis.

##### 2.8.5.1 Urine culture

The medium used, Cysteine Lactose Electrolyte Deficient (CLED), was prepared based on the manufacturer’s instructions. Urine samples were plated using a calibrated 1 mL loop on the CLED agar using the spread method. The inoculated plates were incubated at 35–37 °C within a time period of 18–24 h, after which the growth of bacteria was observed and documented. Gram staining was performed using standard operating procedures on the bacteria isolated to differentiate Gram-negative bacteria from Gram-positive bacteria. The Kirby-Bauer disk diffusion technique on Mueller-Hinton agar was used for the Antibiotic Sensitivity Testing (AST). A standard bacterial suspension, which is identical to the 0.5 McFarland of turbidity, was measured using the nephelometer. The bacterial suspension was evenly spread over the agar surface, and antibiotic disks were placed onto the inoculated agar. Antibiotic disks used for GNB were amikacin, ciprofloxacin, ceftriaxone, gentamicin, cotrimoxazole (trimethoprim-sulfamethoxazole), Augmentin (amoxicillin-clavulanic acid), and tetracycline (based on the WHO standard). Antibiotic disks used for gram-positive bacteria were ciprofloxacin, gentamicin, tetracycline, erythromycin, vancomycin, and cotrimoxazole, based on the WHO standard. After an 18- to 24-h incubation period, the diameter of the inhibition zones was measured in millimeters using a ruler. The findings were reported based on the Clinical and Laboratory Standards Institute (CLSI) or European Committee on Antimicrobial Susceptibility Testing (EUCAST) standards and classified as Sensitive (S), Intermediate (I), or Resistant (R).

##### 2.8.5.2 Blood culture

We dispensed 2–3 mL of venous blood into the bottles of Brain Heart Infusion (BHI) broth, after which they were aerobically incubated at a temperature of 35–37 °C. Daily monitoring of the cultures was done to check for the presence of bacterial growth by observing turbidity or gas formation. Colony isolation was done by subculture on blood agar and MacConkey agar. The identification of isolates was done by Gram staining and the standard biochemical tests, such as the catalase, coagulase, and oxidase tests, and on available API identification systems. The pure isolates were further subjected to AST by the Kirby-Bauer technique on Mueller-Hinton agar. Ceftriaxone, meropenem, vancomycin, gentamicin, ampicillin-clavulanate, and piperacillin-tazobactam were the antibiotics that were used. Measurement of inhibition zones was done and interpreted according to CLSI. Isolates were classified as sensitive, intermediate, or resistant.

##### 2.8.5.3 Routine urine examination

We collected clean-catch midstream urine for the routine urine analysis. The samples were labeled with the unique study code and then examined macroscopically with regard to color, clarity, and odor. Urine chemistry test was done using the dipstick method to identify the presence of blood, leukocytes, nitrites, pH, ketones, specific gravity, protein, and glucose. The samples were centrifuged at 300 rpm for 5–10 min to separate the supernatant from the sediment. A drop of sediment was put on a glass slide, covered with a coverslip, and then observed using a low-power (40x) objective microscope. The presence of bacteria, pus cells, epithelial cells, casts, crystals, and red blood cells was noted. The presence of pus and bacteria revealed the presence of the urinary tract infection (UTI) and was later confirmed by culture.

##### 2.8.5.4 Routine stool examination

We performed stool analysis to identify parasites and other associated infections of the intestines. We then collected samples into sterilized screw-cap containers and labeled them with the study’s unique code. All stool samples were initially examined macroscopically for color, consistency (formed, semi-formed, or watery), and the presence of blood or visible parasites. A small fragment of every specimen was emulsified in 0.9% physiological saline, and one drop of the mixture was put on a slide of clean glass, coupled with a coverslip, and viewed under the light microscope. The objective lens used was a 20x objective lens, which was initially concentrated on larger structures like helminth eggs, larvae, and protozoan cysts, while the 40x objective was used to view smaller structures like protozoan trophozoites, intestinal parasites, red blood cells, and yeast cells. Results were compiled systematically, and positive results were confirmed by a senior scientist.

### 2.9 Data entry and statistical analysis

All data collected were checked for completeness, and each datum was marked with an identification code (WKY-001 to WKY-281). We performed our data analysis using STATA software. Descriptive statistics (frequencies and percentages) were used to describe sociodemographic, clinical, and laboratory parameters. The chi-square tests were used to determine the correlation between categorical variables and the outcome of viral loads. Bivariate logistic regression was used to assess the relationships between each predictor and virological failure. Variables whose *p*-values had a significance of less than 0.05 were used in a multivariate logistic regression, which was run in a backward stepwise selection. Odds ratios were adjusted and had 95% confidence intervals (AOR, CI) used to determine the strength of associations. The p-value was taken as *p* < 0.05.

## 3 Results

### 3.1 Socio-demographic characteristics

Table 1 provides a summary of the outcomes of the treatment based on the demographic and social factors for the 281 people living with HIV(PLHIV) and on sustained ART. There are four groups of virological responses in each category, and the frequencies and percentages are presented as: Target not Detected (TND) < 20 copies/mL, Optimal <200 copies/mL, Suboptimal 200–999 copies/mL, and Virological failures 1,000 copies/mL. Participant ages ranged from 12 to 65 years, with the modal age groups being 30–39 years and 40–49 years (*n* = 100 each, 35.6%). Most participants were females (194, 69.0%). The largest group was those married (*n* = 123, 43.8%), and primary school (*n* = 115, 40.9%) was the highest level of attainment. The majority (43.4% of the participants; *n* = 122) engaged in farming activities while a similar (44.8% of participants; *n* = 43.4) number of Christians were present.

**Table 1:** Socio-demographic characteristics stratified by viral suppression group (*N* = 281).

Variable	Category	TND *n* (%)	Suppressed *n* (%)	Suboptimal *n* (%)	V. failure *n* (%)	Total *n* (%)	*p*-value
ART Duration	TLD	151 (69.0)	10 (4.6)	51 (23.3)	7 (3.2)	219 (100.0)	0.001
	TLE	32 (69.6)	1 (2.2)	11 (23.9)	2 (4.3)	46 (100.0)	
	AZT/3TC/NVP	7 (77.8)	0 (0.0)	2 (22.2)	0 (0.0)	9 (100.0)	
ART duration	1–5 yrs	38 (60.3)	8 (12.7)	11 (17.5)	6 (9.5)	63(100)	0.03
	6–10 yrs	54 (65.9)	9 (10.9)	17 (20.7)	2 (2.4)	82 (100)	
	11–15 yrs	71 (70.3)	14 (13.9)	5 (4.9)	11 (11.0)	101 (100)	
	16–20 yrs	14 (70.0)	2 (10.0)	3 (15.0)	1 (5.0)	20 (100)	
	21–25 yrs	7 (46.7)	3 (20.0)	3 (20.0)	2 (13.3)	15 (2%)	
Defaulted ART	Yes	75 (75.8)	2 (2.0)	20 (20.2)	2 (2.0)	99 (100.0)	0.036
	No	121 (66.5)	11 (6.0)	42 (23.1)	8 (4.4)	182 (100.0)	
Adherence counseling	Yes	187 (69.3)	13(4.8)	61(22.6)	9 (3.3)	270(100.0)	0.068
	No	9 (81.8)	0(0.0)	1(9.1)	1 (9.1)	270 (100.0)	
Herbal medicines	Yes	9 (75.0)	1 (8.3)	2 (16.7)	0 (0.0)	12 (100.0)	0.101
	No	187 (69.5)	12 (4.5)	60 (22.3)	10 (3.7)	269 (100.0)	
Knowledge of VL	Yes	91 (75.2)	2 (1.7)	25 (20.7)	3 (2.5)	121 (100.0)	0.153
	No	105 (65.6)	11 (6.9)	37 (23.1)	7 (4.4)	160 (100.0)	

TLD, Tenofovir/Lamivudine/Dolutegravir; TLE, Tenofovir/Lamivudine/Efavirenz; AZT/3TC/NVP, Zidovudine/Lamivudine/Nevirapine; VL, Viral Load. failure, Virological failure *p < 0.05; **p < 0.01.

### 3.2 Association between antiretroviral therapy (ART) adherence and viral load control

Table 2 presents the correlation between antiretroviral therapy (ART) adherence and viral load suppression. Out of those on TLD treatment, 151 (69.0%) achieved Target Not Detected (TND, undetectable), 10 (4.6%) had optimal suppression, 51 (23.3%) suboptimal suppression, and 7 (3.2%) had virological failure. Similarly, participants on TLE recorded 32 (69.6%) TND, 1 (2.2%) optimal, 11 (23.9%) suboptimal, and 2 (4.3%) failures. Among AZT/3TC/NVP users, 7 (77.8%) achieved TND and 2 (22.2%) were suboptimal. Participants with 11–15 years of therapy had the most TND (71; 70.3%), and participants with 21–25 years on therapy had the lowest TND (7; 46.7%) and the highest suppression failure rates (2; 13.3%). Lower suppression was found in those who defaulted ART (121 = 66.5%) compared to non-defaulters (75 = 75.8%). Adherence counseling showed 187 (69.3%) TND among those counseled, compared with 161 (62.4%) among those in the control group. Moderate differences in viral suppression were observed for herbal medicine use (91; 75.2% vs. 105; 65.6%). Statistically significant factors included ART regimen (*p* = 0.001), ART duration (*p* = 0.030), and ART default history (*p* = 0.036).

**Table 2:** Antiretroviral therapy adherence history among viral load suppression groups (*N* = 281).

Variable	Category	TND *n* (%)	Optimal *n* (%)	Suboptimal *n* (%)	V. failure *n* (%)	Total *n* (%)
Age group	12–19	4 (80.0)	0 (0.0)	1 (20.0)	0 (0.0)	5 (100)
	20–29	24 (68.6)	2 (5.7)	8 (22.9)	1 (2.9)	35 (100)
	30–39	71 (71.0)	7 (7.0)	19 (19.0)	3 (3.0)	100 (100)
	40–49	72 (72.0)	1 (1.0)	23 (23.0)	4 (4.0)	100 (100)
	50–65	24 (60.0)	3 (7.5)	12 (27.5)	2 (5.0)	41 (100)
Sex	Male	48 (55.2)	15 (17.2)	19 (21.8)	5 (5.8)	87 (100)
	Female	101 (52.1)	26 (13.4)	57 (29.4)	10 (5.1)	194 (100)
Marital status	Married	90 (73.2)	6 (4.9)	22 (17.8)	5 (4.1)	123 (100)
	Single	41 (77.4)	1 (1.9)	9 (17.0)	2 (3.7)	53 (100)
	Divorced	40 (76.9)	2 (3.8)	9 (17.4)	1 (1.9)	52 (100)
	Widowed	24 (45.3)	4 (7.5)	23 (43.4)	2 (3.8)	53 (100)
Educational status	Tertiary	42 (72.4)	3 (5.2)	12 (20.7)	1 (1.7)	58 (100)
	SHS	46 (76.7)	3 (5.0)	9 (15.0)	2 (3.3)	60 (100)
	Primary	75 (65.2)	4 (3.5)	30 (26.1)	6 (5.2)	115 (100)
	None	33 (68.8)	3 (6.3)	11 (22.9)	1 (2.1)	48 (100)
Occupation	Farmer	72 (74.2)	3 (3.1)	17 (17.7)	2 (2.1)	97 (100)
	Trader	43 (81.1)	0 (0.0)	9 (17.0)	1 (1.9)	53 (100)
	Teacher	17 (65.4)	1 (3.8)	6 (23.1)	1 (3.8)	25 (100)
	Others	61 (57.5)	9 (8.5)	30 (28.3)	6 (5.7)	106 (100)
Religious status	Christians	82 (67.2)	11 (9.0)	25 (20.5)	4 (3.3)	122 (100)
	Muslims	45 (48.9)	17 (18.5)	20 (21.7)	10 (10.9)	92 (100)
	Traditionalist	39 (58.2)	12 (17.9)	14 (20.9)	2 (3.0)	67 (100)
District	K. N. East	81 (64.3)	13 (10.3)	25 (19.8)	7 (5.7)	126 (100)
	K. N. West	44 (75.8)	2 (3.5)	9 (15.5)	3 (5.3)	58 (100)
	Builsa North	58 (59.8)	10 (10.3)	25 (25.8)	4 (4.1)	97 (100)

TND, Target Not Detected; V. Failure, Virological Failure; K. N., Kasena-Nankana; SHS, Senior High School.

### 3.3 Prevalence of viral load suppression rate among HIV- positive participants

**Figure 1:**
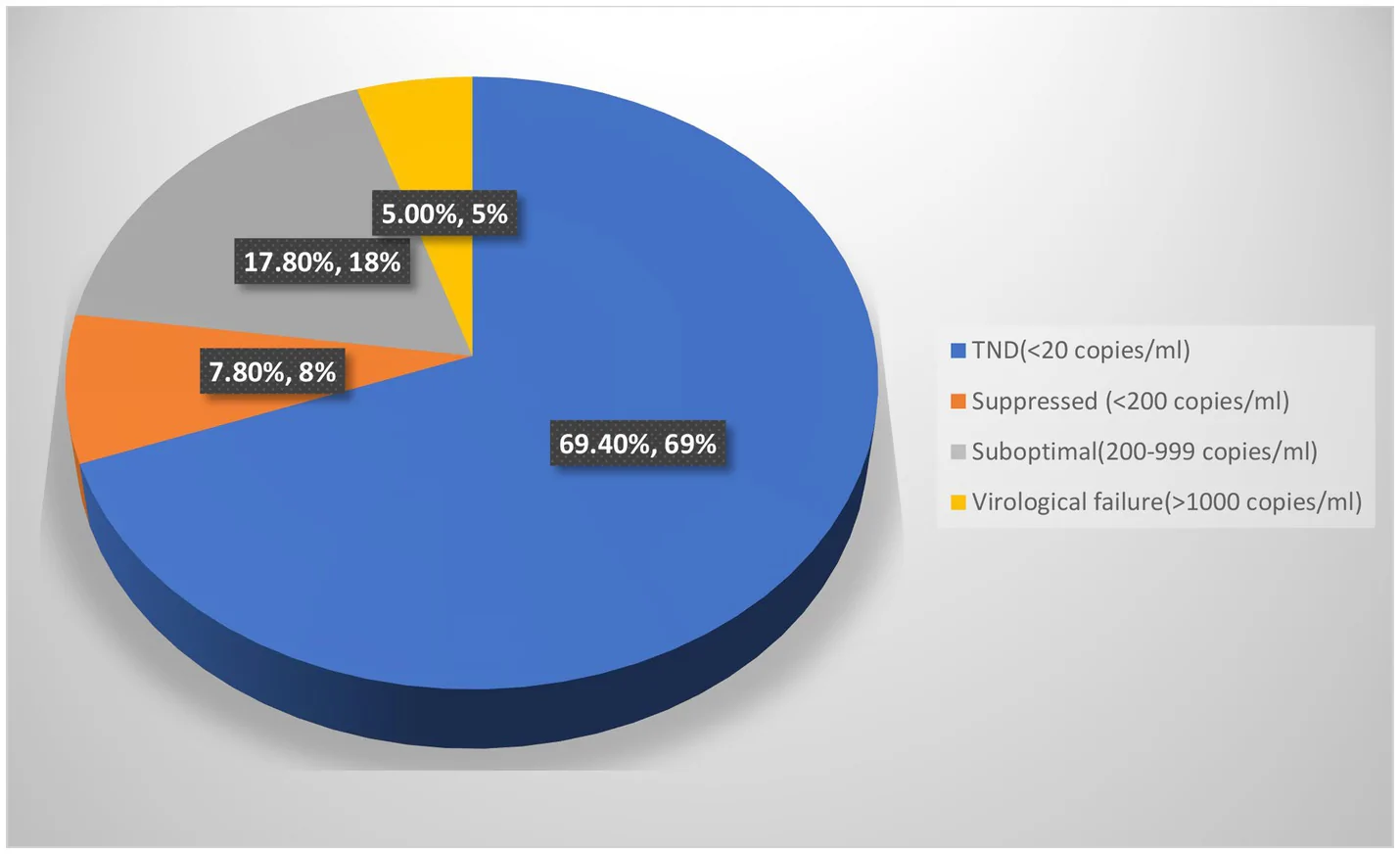
Viral load suppression rates among HIV-positive participants.

Among the 281 Participants under antiretroviral therapy (ART), most of them (69.4%) had full viral suppression with the viral loads of them being undetected (TND). (7.8%) had optimal viral loads less than 200 copies/ml. However, (17.8%) had suboptimal viral suppression (200–999 copies/ml); and (5.0%) had virologic failure and viral loads ≥1,000 copies/ml (Figure 1).

### 3.4 Association between systemic co-infections and viral load suppression groups

Table 3 present virological outcomes by bacteremia, UTI and GIT infection. The percentage of study participants who achieved TND was also higher in the bacteremia (69.0%) than in the non-bacteremia (69.5%) group, Virological failure was higher in the bacteremia group (8.5%) than the non-bacteremia group (0.5%). Majority of those with UTI achieved undetectable viral loads of 186(73.9%) as compared to those with UTI, 17(54.8%). With virological failure, 3(11.8%) had UTI and 2(0.8%) had no UTI. Among 104 participants with GIT (73, 69.6%) achieved TND while 5(4.8%) had virological failure. For those without GIT (177), most of the subjects (122, 68.9%) had TND viral loads with 2(1.1%) virologic failure. No statistical relationship was observed in bacteremia (*p* = 0.300), UTI (*p* = 0.076) and GIT (*p* = 0.371).

**Table 3:** Distribution of systemic infections across viral suppression groups (*N* = 281).

Co-infection	Status	TND *n* (%)	Optimal *n* (%)	Suboptimal *n* (%)	V. failure *n* (%)	*p*-value
Bacteraemia	No (*n* = 210)	146 (69.5)	16 (7.6)	47 (22.4)	1 (0.5)	0.300
	Yes (*n* = 71)	49 (69.0)	6 (8.5)	10 (14.1)	6 (8.5)	
UTI	No (*n* = 252)	186 (73.9)	16 (6.3)	48 (19.0)	2 (0.8)	0.076
	Yes (*n* = 29)	17 (54.8)	3 (9.7)	9 (31.0)	3 (11.8)	
GIT	No (*n* = 177)	122 (68.9)	14 (7.9)	39 (22.0)	2 (1.1)	0.371
	Yes (*n* = 104)	73 (69.6)	8 (7.7)	18 (17.3)	5 (4.8)	

TND, Target Not Detected; GIT, Gastrointestinal Tract, UTI-Urinary tract infection.

### 3.5 Clinical characteristics of the participants

#### 3.5.1 Association between liver function biomarkers and viral Lod suppression

Table 4 presents the biochemical association between LFT Indicators and Viral Load Suppression. Total bilirubin (*p* = 0.796); conjugated bilirubin (*p* = 0.831); hepatic enzymes (AST *p* = 0.817; ALT p 0.838; GGT *p* = 0.644; ALP *p* = 0.777) were non-significant. Total protein was significantly associated with viral suppression (*χ*^2^ = 8.23, *p* = 0.042) and also A/G ratio was significantly associated with suppression (*χ*^2^ = 9.08, *p* = 0.029).

**Table 4:** Association between LFT indicators and viral load suppression (*N* = 281).

Variable	Category	TND *n* (%)	Suboptimal *n* (%)	Optimal *n* (%)	V. failure *n* (%)	Total	*χ*^2^ (df)	*p*-value
Bilirubin (Total) μmol/	Normal (≤21)	182 (70.5)	56 (21.7)	18 (7.0)	2 (0.8)	258	1.02	0.796
	Elevated (>21)	16 (69.6)	5 (21.7)	2 (8.7)	0	23		
Bilirubin (Conjugated/ μmol/L)	Normal (≤5)	176 (70.1)	55 (21.9)	17 (6.8)	3 (1.2)	251	0.88	0.831
	Elevated (>5)	22 (73.3)	6 (20.0)	2 (6.7)	0 (0.0)	30		
AST(U/L)	Normal (≤40)	177 (67.8)	57 (21.9)	22 (8.4)	5 (1.9)	261	0.94	0.817
	Elevated (>40)	15 (75.0)	4 (20.0)	1 (5.0)	0 (0.0)	20		
ALT(U/L)	Normal (≤50)	178 (69.0)	54 (20.9)	21 (8.1)	5 (1.9)	258	0.85	0.838
	Elevated (>50)	15 (65.2)	7 (30.4)	1 (4.3)	0 (0.0)	23		
GGT(U/L)	Normal (≤60)	160 (68.4)	50 (21.4)	21 (9.0)	4 (1.2)	235	1.67	0.644
	Elevated (>60)	32 (69.6)	11 (23.9)	2 (4.3)	1 (2.2)	46		
ALP(U/L)	Normal (≤120)	174 (69.9)	55 (22.1)	18 (7.2)	2 (0.8)	249	1.10	0.777
	Elevated (>120)	24 (75.0)	6 (18.8)	2 (6.2)	0	32		
T. Protein (g/L)	Norm. (60–88)	120 (72.2)	30 (18.1)	10 (6.1)	6 (3.6)	166	8.23	0.042
	Abn.(>88 or<60)	75 (65.2)	34 (29.6)	3 (2.6)	3 (2.6)	115		
Albumin (g/l)	Norm. (35–55)	140 (70.)	40 (20.0)	12 (6.0)	8 (4.0)	200	6.52	0.087
	Abn (>55or < 35)	53 (65.5)	24 (29.6)	1 (1.2)	3 (3.7)	81		
Globulin	Norm. (20–35)	130 (71.0)	38 (20.8)	10 (5.5)	5 (2.7)	183	6.98	0.064
	Abn.(>35 or<20)	64 (65.3)	25 (25.5)	3 (3.1)	5 (5.1)	98		
A/G Ratio	Norm. (1.0–1.8)	135 (73.0)	35 (18.9)	10 (5.4)	5 (2.7)	185	9.08	0.029
	Abn. (<1.0 or>8)	60 (62.5)	28 (29.2)	3 (3.1)	5 (5.2)	96		

Norm, Abn, Abnormal; TND, Target Not Detected; V. failure, Virological failure; AST, Aspartate aminotransferase; ALT, Alanine aminotransferase; GGT, Gamma Glutamyl Transferase; T. Protein, Total Protein.

#### 3.5.2 Association between renal function indicators and viral load suppression

All *p* values were > 0.05, indicating that renal function parameters did not show any statistically significant associations with the virological outcomes. For elevated urea levels (U > 11.1 mmol/L), however, a higher proportion of virological failure (37.5%) was found and a corresponding decrease in TND (37.5%). The small size of this subgroup (*n* = 8) should be noted in this regard.

#### 3.5.3 Association between METS indicators and viral load suppression

From Table 5, among those with normal systolic blood pressure, TND viral load was observed in 60 (66.7%) participants, compared to 86 (68.8%) with elevated and 43 (65.2%) with hypertension (*p* = 0.990). No differences were seen in TND viral load among participants with normal and elevated waist circumference and among those with normal and high-risk waist circumference (*p* = 0.946). No significant differences were observed for TND viral loads across BMI categories among the underweight (25, 62.5%), normal-weight (139, 68.5%), overweight (19, 70.4%), and obese (7, 63.6%) participants (*p* = 0.982).

**Table 5:** Association between METS indicators and viral load suppression (*N* = 281).

Variable	Category	TND n (%)	Suboptimal n (%)	Optimal n (%)	V.failure n (%)	Total	χ^2^	p-value
Sys. BP (mmHg)	Normal (<120)	60 (66.7)	21 (23.3)	7 (7.8)	2 (2.2)	90	0.8	0.99
	Elevated (120–139)	86 (68.8)	24 (19.2)	11 (8.8)	4 (3.2)	125		
	Hypertensive (≥140)	43 (65.2)	16 (24.2)	5 (7.6)	2 (3.0)	66		
Lipidaemia (mg/dl)	Desirable (<200)	191 (68.2)	61 (21.8)	23 (8.2)	5 (1.8)	280	0.5	0.915
	High (≥240)	0 (0.0)	0 (0.0)	0 (0.0)	1 (100.0)	1		
RBS (mmol/L)	Normal (<140)	185 (69.2)	56 (20.9)	21 (7.9)	5 (2.0)	267	0.4	0.912
	Diabetic (≥200)	6 (42.9)	5 (35.7)	2 (14.3)	1 (7.1)	14		
Waist Circ. (cm)	Normal (<80)	54 (71.1)	15 (19.7)	6 (7.9)	1 (1.3)	76	0.9	0.946
	Elevated (80–93)	80 (67.2)	25 (21.0)	11 (9.2)	3 (2.5)	119		
	High risk (≥94)	57 (66.3)	21 (24.4)	6 (7.0)	2 (2.3)	86		
BMI (kg/m2)	Underweight (<18.5)	25 (62.5)	11 (27.5)	3 (7.5)	1 (2.5)	40	1.9	0.982
	Normal (18.5–24.9)	139 (68.5)	43 (21.2)	18 (8.9)	3 (1.5)	203		
	Over wt. (25–29.9)	19 (70.4)	6 (22.2)	1 (3.7)	1 (3.7)	27		
	Obese (≥30)	7 (63.6)	2 (18.2)	1 (9.1)	1 (9.1)	11		

BMI, Body Mass Index; RBS, Random Blood Sugar; Sys. BP, Systolic Blood Pressure; Waist Circ, Waist Circumference; TND, Target not Detected; V. Failure, Virological failure.

#### 3.5.4 Overall viral load suppression outcomes stratified by liver function, renal function, and MS status

**Figure 2:**
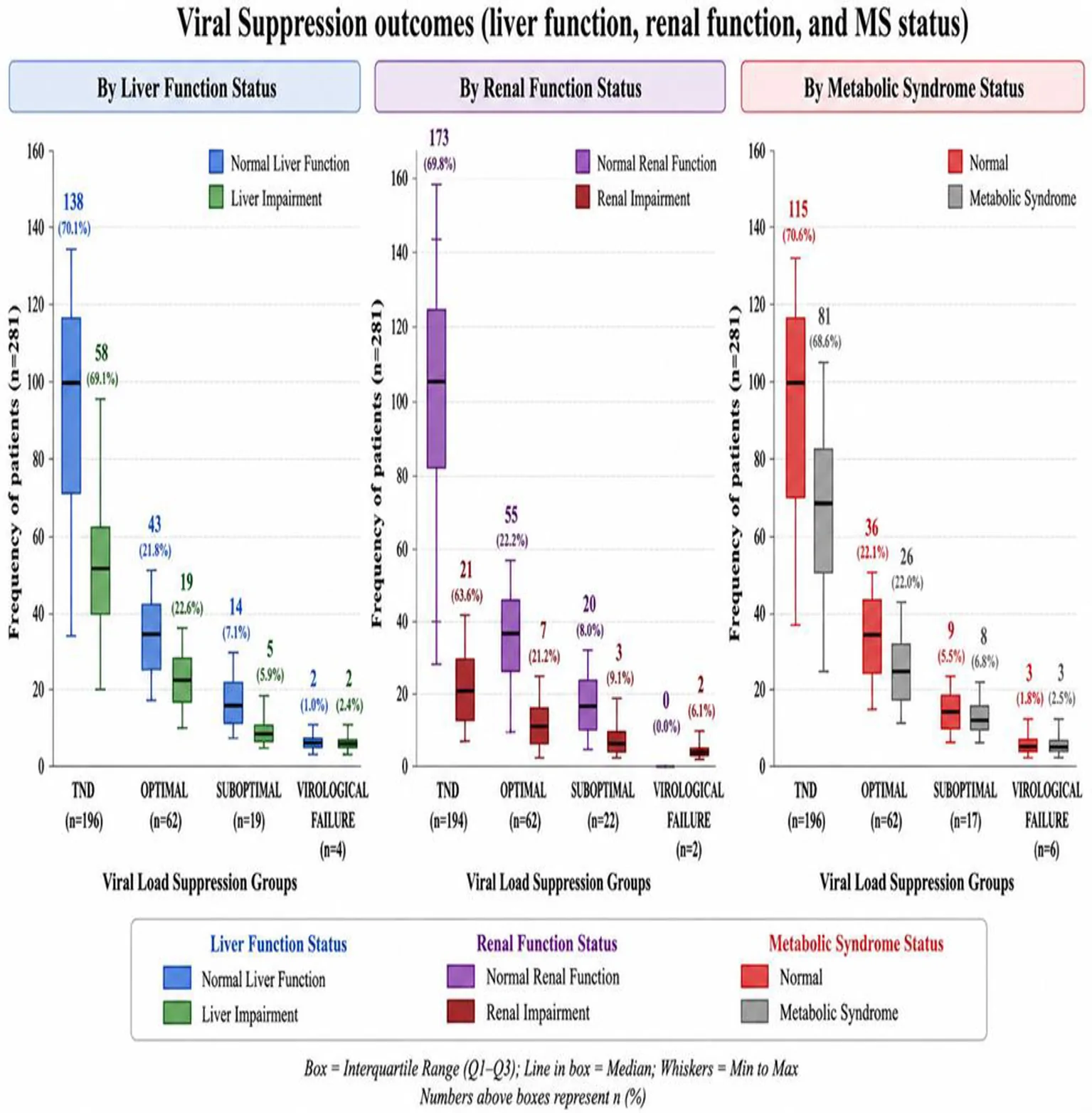
Association between viral suppression outcomes with hepatic dysfunction, renal impairment, and metabolic syndrome among HIV/AIDS patients on sustained antiretroviral therapy.

The box-and-whisker plots below shows the results of viral load suppression (by clinical category). For liver function status, normal liver function shows TND = 138 (70.1%), optimal = 43 (21.8%), suboptimal = 14 (7.1%), and virological failure = 2 (1.0%), while liver impairment shows TND = 58 (69.1%), optimal = 19 (22.6%), suboptimal = 5 (5.9%), and virological failure = 2 (2.4%). For renal function status, normal renal function shows TND = 173 (69.8%), optimal = 55 (22.2%), suboptimal = 20 (8.0%), and virological failure = 0 (0.0%), while renal impairment shows TND = 21 (63.6%), optimal = 7 (21.2%), suboptimal = 3 (9.1%), and virological failure = 2 (6.1%). For metabolic syndrome status, the normal group shows TND = 115 (70.6%), optimal = 36 (22.1%), suboptimal = 9 (5.5%), and virological failure = 3 (1.8%), while the metabolic syndrome group shows TND = 81 (68.6%), optimal = 26 (22.0%), suboptimal = 8 (6.8%), and virological failure = 3 (2.5%) (Table 6; Figure 2).

**Table 6:** Association between renal function parameters and viral load suppression.

Variable	Category	TND *n* (%)	Suboptimal *n* (%)	Optimal *n* (%)	V. failure *n* (%)	Total	*χ*^2^ (df)	*p*-value
Creatinine (mmol/L)	Normal (≤135)	162 (66.9)	53 (21.9)	25 (10.3)	2 (0.9)	242	2.0	0.564
	Elevated (>135)	27 (69.2)	9 (23.1)	2 (5.1)	1 (2.6)	39		
Urea (mmol/L)	Normal (≤11.1)	190 (69.6)	61 (22.3)	20 (7.3)	2 (0.8)	273	1.3	0.708
	Elevated (>11.1)	3 (37.5)	1 (12.5)	1 (12.5)	3 (37.5)	8		
BUN (mmol/L)	Normal (≤20)	182 (68.7)	60 (22.6)	21 (7.9)	2 (0.8)	265	2.8	0.412
	Elevated (>20)	12 (75.0)	2 (12.4)	1 (6.3)	1 (6.3)	16		
eGFR	Normal (≥90)	173 (69.5)	55 (22.1)	19 (7.6)	2 (0.8)	249	1.6	0.366
	Reduced (<90)	21 (65.6)	7 (21.9)	3 (9.4)	1 (3.1)	32		
Sodium (mmol/L)	Normal (135–145)	188 (70.4)	59 (22.1)	18 (6.7)	2 (0.8)	267	1.7	0.644
	Abn. (<135/ >145)	7 (50.0)	3 (21.4)	3 (21.4)	1 (7.2)	14		
Potassium (mmol/L)	Normal (3.5–5.1)	186 (70.2)	59 (22.2)	18 (6.8)	2 (0.8)	265	1.8	0.624
	Abn. (<3.5 />5.1)	9 (56.2)	3 (18.8)	4 (25.0)	0 (0.0)	16		
Chloride (mmol/L)	Normal (98–107)	189 (70.0)	60 (22.2)	18 (6.7)	3 (1.1)	270	1.9	0.581
	Abn. (<98/ >107)	5 (45.5)	4 (36.3)	2 (18.2)	0 (0.0)	11		

Abn, Abnormal; eGFR, Estimated Glomerular Filtrate Rate; TND, Target Not Detected; V. Failure, Virological failure; BUN, Blood Urea Nitrogen. All *p*-values > 0.

#### 3.5.5 Association between hematological parameters and viral load status

Virological outcome was significantly associated with hemoglobin (*p* = 0.048), hematocrit (*p* = 0.046), lymphocytes (*p* = 0.034), MCHC (*p* = 0.029), and neutrophils (*p* = 0.018). There were 59.3% of participants in whom neutrophils were low with TND (59.4) %, whereas 73.7% of TND had a normal neutrophils TND (*χ*^2^ = 10.12; p = 0.018). Neutropenia was the best hematology predictor. There was no significant association in WBC count (*p* = 0.096) and platelets (*p* = 0.117; Table 7.

**Table 7:** Association between hematological indicators and viral load suppression groups.

Variable	Category	TND *n* (%)	Suboptimal *n* (%)	Optimal *n* (%)	V. failure *n* (%)	Total *n* (%)	*χ*^2^ (df)	*p*-value
Hemoglobin g/dl	Normal (11.5–16.0)	130 (72.2)	30 (16.7)	12 (6.7)	8 (4.4)	180	7.91	0.048
	Anaemic (<11.5)	64 (63.4)	32 (32.7)	2 (1.9)	2 (1.9)	101		
Hematocrit (HCT)%	Normal (36.0–48.0)	129 (72.5)	31 (17.4)	12 (6.7)	6 (3.4)	178	7.98	0.046
	Abn. (<36.0/>48.0)	65 (63.1)	32 (31.1)	2 (1.9)	4 (3.9)	103		
WBC Count	Normal (4.0–10.0)	140 (70.7)	38 (19.2)	12 (6.1)	8 (4.0)	198	6.34	0.096
X10^9^/L	Leukopenia (<4.0)	54 (65.0)	25 (30.1)	2 (2.4)	2 (2.4)	83		
Platelets	Normal (100–300)	145 (71.4)	38 (18.7)	12 (5.9)	8 (3.9)	203	5.88	0.117
X10^9^/L	Thrombocytosis (>300)	50 (64.1)	25 (32.1)	2 (2.6)	1 (1.3)	78		
Lymphocytes %	Normal (20.0–40.0)	135 (74.2)	30 (16.5)	10 (5.5)	7 (3.8)	182	8.67	0.034
	Lymphocytopenia (<20.0)	61 (61.7)	34 (34.3)	2 (2.0)	2 (2.0)	99		
-MCHC g/dl	Normal (32.0–36.0)	144 (73.5)	34 (17.3)	12 (6.1)	6 (3.1)	196	9.02	0.029
	Abnormal (<32.0)	52 (62.7)	30 (36.1)	2 (2.4)	1 (1.2)	85		
Neutrophils %	Normal (50.0–69.0)	140 (73.7)	32 (16.8)	12 (6.3)	6 (3.2)	190	10.12	0.018
	Neutropenia (<50.0)	54 (59.3)	31 (34.1)	2 (2.2)	4 (4.4)	94 (1)		

MCHC, Mean Corpuscular Hemoglobin Concentration; WBC, White Blood Cell. *p < 0.05.

#### 3.5.6 Logistic regression model of associated factors influencing suboptimal

Table 8 shows the logistic regression model indicating socio-demographic and clinical factors associated with suboptimal viral suppression among 281 HIV clients on sustained ART. Being male was marginally associated with lower odds of suppression than females (AOR = 0.91, *p* = 0.042). The participants of single marital status had lower odds than married participants (AOR = 0.71, *p* = 0.063). Poor suppression (AOR = 0.89, *p* = 0.037) was associated with comorbidities like tuberculosis and hypertension. Patients who reported infections had higher risk (AOR = 0.76, *p* = 0.041). Those living more than 5 km from the health facility had reduced odds of suppression (AOR = 0.68, *p* = 0.008). Easy access to healthcare, on the other hand, did have a positive impact on outcome (AOR = 1.52, *p* = 0.004). Stigma and cultural/religious influence negatively affected suppression (AOR = 0.66, *p* = 0.003 and AOR = 0.71, *p* = 0.015, respectively). Non-significant variables were the water source, and other treatments.

**Table 8:** Logistic regression model of associated factors influencing suboptimal viral suppression (*N* = 281).

Variable	Category	Ref.	*p*-value	COR (95% CI)	AOR (95% CI)
Sex	Female	Male	0.042	0.94 (0.72–1.22)	0.91 (0.69–1.20)
Marital status	Married	Single	0.063	0.76 (0.58–0.99)	0.71 (0.53–0.95)
Comorbidities (TB, HTN, etc.)	No	Yes	0.037	0.67 (0.70–1.18)	0.89 (0.68–1.17)
Told had infection	Yes	No	0.041	0.79 (0.63–0.99)	0.76 (0.59–0.98)
On other treatment	Yes	No	0.16	0.84 (0.66–1.07)	0.82 (0.63–1.07)
Source of drinking water	Safe/Pipe	Unsafe	0.32	1.12 (0.89–1.41)	1.09 (0.86–1.38)
Treat drinking water	Yes	No	0.11	1.21 (0.95–1.54)	1.18 (0.92–1.52)
Distance to health facility	>5 km	≤5 km	0.008	0.73 (0.58–0.92)	0.68 (0.52–0.89)
Easy access to healthcare	Yes	No	0.004	1.45 (1.12–1.88)	1.52 (1.17–1.98)
Experienced stigma	Yes	No	0.003	0.69 (0.54–0.88)	0.66 (0.50–0.86)
Cultural/religious influence	Yes	No	0.015	0.74 (0.58–0.94)	0.71 (0.55–0.93)

COR, Crude Odds Ratio; AOR, Adjusted Odds Ratio; CI, Confidence Interval. Bold *p*-values indicate statistical significance (*p* < 0.05). Variables with *p* > 0.05 after full multivariate adjustment were not retained as independent predictors.

## 4 Discussion

### 4.1 Socio-demographic factors

The socio demographic profile of participants in this study mirrors the typical HIV treatment population in resource limited settings, where the epidemic disproportionately affects economically active adults. The majority of cases were concentrated in the age groups of 30–39 years and 40–49 years, which reflects the national and regional prevalence of HIV in the productive age in sub–Saharan Africa (16, 17). While there were no noted statistically significant association between age and virological suppression, the median age of 50–65 years was associated with higher rates of suboptimal suppression and virological failure, consistent with evidence that HIV aging is complicated by comorbidities, polypharmacy, and treatment fatigue, which have indirect effects on adherence and long-term health outcomes (18). The high proportion of female respondents is an epidemiology fact due to the higher involvement of women with health system in healthcare for maternal and reproductive health services in sub-Saharan countries (19). Sex was not a standalone predictor of viral suppression, but women’s attendance at the clinic differed from men’s, who would seek care later and could be influenced by rigid gender norms and stigma. For retention in care, adherence behavior, regimen continuity, and access to care are more important than sex differences in the context of achieving virological outcomes (20). There were parallels in the socio-economic indicators. Participants that were married showed greater viral suppression, further revealing the benefits of stable interpersonal relationships. Adherence is increased by emotional support and shared responsibility provided by a supportive spouse, while widowhood can trigger psychological distress, social isolation, and financial burden that can affect adherence (21). Higher education levels were associated with higher levels of health literacy and communication with providers and made the greatest progress in viral suppression. The lack of a statistical significance indicates that although education is important, it does not compensate for structural factors including access to food, transportation, and poverty that disrupt a commitment to sustained involvement in care (22). There were also differences in occupational patterns, indicating economic differences. Traders had the highest suppression rates, having the financial flexibility and ability to distribute indirect care costs, such as travel and nutrition. Yet, farmers and informal sector workers faced suboptimal suppression because of unstable incomes and seasonal employment needs (23). Religious affiliation and district location did not differ, suggesting that psychosocial factors play a mediating role, and structural inequities related to facility location are still significant factors affecting treatment outcomes (24).

### 4.2 Antiretroviral therapy adherence history

Our analysis of ART (Adherence) History highlights the challenge of making the best use of the ARVs in relation to the duration of treatments and adherence to behavior to ensure that treatment is successful in controlling the HIV virus. Our analysis revealed that virologic outcomes are better with modern integrase strand transfer inhibitor (INSTI)–based regimens, including those containing Tenofovir/Lamivudine/Dolutegravir (TLD), with almost 69% having undetectable viral loads. These findings are consistent with international data that shows that Dolutegravir has a greater genetic barrier to resistance and is more tolerated than older NNRTI-containing combinations like Tenofovir/Lamivudine/Efavirenz (25). Older treatment regimens such as AZT/3TC/NVP still suppressed small numbers of subjects, but the potential for the long-term development of resistance mutations is well established and known (26). A nonlinear trajectory was found for treatment duration. Highest viral suppression was among those on ART for 11–15 years, marking some level of ART resilience and adherence by long-term survivors. However, in those treated for more than 20 years, viral suppression was decreased and virological failure increased to 13.3%. This agrees with other studies of older HIV populations, with treatment fatigue, burdens of multiple toxicities and archival resistance mutations all having a negative impact on adherence and outcomes (27). These findings highlight the importance of more robust follow-up and treatment optimization in long-term treated patients (lifetime treatment). Outcomes were further modified with compliance for adherence behavior. Defaulters paradoxically had higher rates of their viruses being undetected than no defaulters; this is likely because of intensive counseling and reengagement efforts after lapses of treatment, as has been described in earlier contexts (28, 29). Clinic attendance does not imply that the client “is adherent” with silent non-adherence among “non-defaulters” (20). Counseling and viral load literacy had positive trends; thus, patients who received counseling and those who had a conceptual understanding of viral load were more likely to suppress their viral load, demonstrating that behaviors can influence suppression and adherence (21). Herbal medicine use did not significantly disrupt viral suppression (*p* = 0.101), with 75.0% of users achieving an undetectable viral load (TND) compared to 69.5% of non-users. This observation, however, is constrained by the small number of patients used (*n* = 12) and by the potential for interactions with other herbs commonly used by patients. Therapeutic pluralism in which patients take both ARTs and herbal treatment at the same time is common in West Africa (4). These preliminary statistics are positive, but clinical monitoring of herbal use needs to be ongoing; larger numbers of patients often expose adherence issues and/or toxicity that can compromise long-term virological outcomes.

### 4.3 Prevalence of viral suppression rates among HIV/AAIDS patient on ART

Our findings from the study reflect advancement and continued progress toward best treatment outcomes in Kasena Nankana and Builsa municipalities in the upper east region of Ghana. While clinically meaningful reductions in viral rebounds occurred in the majority of people in long-term care, a significant number did not achieve complete viral suppression, low-level viremia or virological failure. A similar study in the Upper East Region, which recorded an estimated suppression rate of around 51%, revealed experience with programmatic changes over the past few years that may be due to the introduction of dolutegravir-based regimens and improved clinical monitoring (6). This indicates that there are regional variations, and improvements in HIV monitoring programs over the last few years. The suppression rate from the study was comparable with other facility-based studies conducted in Ghana, which reported 75.9% suppression rate in the Western Region of Ghana (30). While similar findings in a secondary and tertiary hospital conducted between 2019 and 2023 revealed suppression rates of from 73.8 and 74.2%, respectively (31). However, the consistency implies that the observed outcomes are not simply related to facility-level issues, but rather to a wider system of issues (30). The significant improvement in viral suppression rates that was observed in the present study compared to past evaluations demonstrates a successful transformation resulting from clinical, institutional and structural enhancements within the regional health system. While these achievements have been made, it seems that unsuppressed subpopulations of HIV can still be found within the country, mostly as a result of poor adherence, gaps in viral load testing, drug interactions, drug malabsorption, and emerging drug resistance.

### 4.4 Microbiological systemic co-infections: intestinal, urinary, and blood infections

The investigation also revealed that there was a strong link between the microbiological co-infections and the poor virological outcomes even though no statistically significant association was established. Higher rates of suboptimal viral suppression and treatment failure were found among the participants with gastrointestinal parasitic infections, urinary tract infections, and bloodstream infections. Chronic immune activation and associated inflammatory cytokine release occurs when experiencing active infections, that can reactivate latent HIV replication and lower the effectiveness of ART (32). Besides, there are localized infections within the gut which lead to alteration of the architecture of the enterocytes and of the gut associated lymphoid tissue (GALT) (33). This confined inflammation of the mucosal tissues results in substantial drug malabsorption and nutritional deficits that may make it impossible for antiretroviral medicines to obtain the optimal therapeutic levels of the medicines in the blood (34). Similar observations to enteric parasitic infection and chronic gastrointestinal inflammation have been consistently reported in HIV infected individuals in Sub-Saharan Africa (SSA), which revealed that gastrointestinal infections can have a deleterious effect on gut mucosal integrity and impair nutrient and drug absorption (35, 36). These co-infections were not significant in analysis, but are important indicators of physiological stress and clinical vulnerability. These findings are consistent with the emerging knowledge that HIV care goes beyond using ART. For long-term viral suppression, comprehensive and coordinated clinical care is necessary, including early and efficient diagnosis and treatment of OIs, routine clinical checks for other systemic conditions, and regular monitoring of organ function. Coexisting infections, immune activation, and chronic inflammation can complicate recovery of the immune system, drug absorption and treatment sustainability (37).

### 4.5 Association between metabolic syndrome and virological outcomes

No statistically significant relationship is observed for the detailed analysis of the components of metabolic syndrome (random blood sugar, blood pressure, lipid profiles, waist circumference, and BMI) when compared with viral load suppression. Though a significant proportion of them had disordered blood pressure, central obesity or overweight status, these health challenges did not reduce the biological effectiveness of the regimen of antiretroviral medications (ART). Although “metabolic syndrome” is more commonly associated with cardiovascular and macrovascular risk over the long term, it did not appear to impede virological suppression in the short term (38). Pathophysiologically, this dissociation reflects the fact that the cellular-enzymatic components of HIV replication and the mechanism of action of current antiretroviral therapies are not on the same axis of peripheral lipid deposition, insulin signal transduction or endothelial wall stress (39). The findings are consistent with previous regional study from West Africa and extensive global literature that therapy-acquired metabolic changes are independent of the virus metabolism (40). The present study indicates that, even in the context of a high prevalence of metabolic syndrome from long-term exposure to drugs, metabolic syndrome does not hinder the clearance of viruses but acts as a concurrent non-communicable disease. Ultimately, adherence to behavior, and the effectiveness of the drug treatment, are what really matters in deciding whether or not the therapy is working (41). Therapeutic failures should also be differentiated from metabolic comorbidities in a programmatic way from a clinical standpoint. Emphasis should be placed on intensive adherence counseling and ongoing monitoring of viruses in addition to ongoing management of metabolic abnormalities along with multiple concurrent chronic disease pathways.

### 4.6 Association between liver function status and viral load suppression

Biochemical assessment of liver function during chronic treatment with antiretroviral therapy (ART) is multilayered and presents a clear relationship to virological efficacy. Treatment outcomes strongly correlated with the markers of hepatic synthetic capacity such as serum total proteins and the albumin- globulin (A/G) ratio. However, no correlation was observed for serum enzymes: AST, ALT, alkaline phosphatase, gamma-glutamyl transferase and also, for bilirubin fractions. These are independent variables and are very good surrogate markers for sub-optimal viral suppression and complete treatment failure. In this disclosure this liver synthetic function is indeed a far more accurate means to follow on to virological findings than local liver damage (42). Abnormalities in these synthetic markers usually reflect underlying deep physiological abnormalities such as chronic microinflammation, severe immune dysregulations and underlying protein-energy malnutrition, all of which directly deprive complete immune recovery (43). In chronic systemic viral infections, excess microbial translocation through the leaky gut resulting from unchecked viral replication leads to chronic, inflammatory response of the gut microbiota attributed to lipopolysaccharide (LPS) surge in the portal system (44, 45). This continued inflammation leads to the formation of pro-inflammatory cytokines which have an effect on hepatic protein synthesis to raise inflammatory globulins instead of albumins (44). This biochemical profile is well aligned with the international literature which has demonstrated that the combination of synthetic liver markers and variation in the plasma protein profile is superior to individual transaminase elevations and serve as very sensitive markers of chronic immune exhaustion (46, 47). From a clinical standpoint, there should be programs of regular monitoring of A/G ratios and total protein which are extremely valuable for resource-limited health systems. Microinflammation, severe immune dysregulations and underlying protein-energy malnutrition, all of which directly deprive complete immune recovery. In terms of clinical value to resource-limited health systems, routine monitoring of A/G ratio and TP measurements are extremely valuable.

### 4.7 Association between renal function status and viral load suppression rates

Our findings showed that there was no meaningful correlation between renal biomarkers such as serum creatinine, urea, blood urea nitrogen (BUN) and estimated glomerular filtration rate (eGFR) with the viral load suppression. Likewise, there were no significant correlation between serum electrolyte concentrations (sodium, potassium and chloride) and virological outcomes. About one third of the participants had increased levels of renal retention markers or mild electrolyte disturbances, but this did not affect virologic response to treatment. This suggests that renal and electrolyte disturbances do not directly and immediately affect mechanisms of viral suppression in HIV ptients (48). In line with present findings in Ghana, renal impairment commonly occurred in people on ART and that this was attributed to the presence of long-term metabolic comorbidities (e.g., hypertension and diabetes) and long-term exposure to potentially nephrotoxic ARTs rather than to the presence of ongoing active viral replication (49). The problem of kidney dysfunction in this population is not absolute failure of the drugs, but a multifaceted, results of old age, chronic comorbidities and drug toxicities. In addition, mild renal insufficiency and electrolyte disturbances have been shown not to affect virological failure (50). These results highlight the need to carefully distinguish treatment safety monitoring from virological monitoring because treatment-related complications like renal impairment may be present without any active viral replication or virological failure. Although there is no direct correlation between the measurements of viral load and renal and electrolyte, these are considered as one of the important parameters of HIV management. The various widely-used antiretroviral drugs have individual characteristic nephrotoxic characteristics of their own use, for example tenofovir disoproxil fumarate (TDF) (51). Long-term exposure can cause proximal renal tubular dysfunction, electrolyte wasting and CKD (50). Overweight and hydrated state of the kidney is a common early sign of renal stress caused by drugs (52). One essential measure of preventative care is the regular check-up to monitor electrolytes and renal function.

### 4.8 Association between hematological status viral load suppression

The associations between viral load suppression status and hematological abnormalities were particularly strong with respect to poor virological outcomes. The prevalence of anemia was quite high and was associated with suboptimal viral suppression and virological failure. Anemia of chronic disease is caused by chronic, uncontrolled replication of HIV, which leads to ongoing immune activation and inflammatory cytokine production, causing iron metabolism abnormalities and diminished bone marrow activity (53). The decreased oxygen-carrying ability can inhibit immune responses and make it harder to effectively control the replicating viruses. Similar findings have been reported in African research where anemia was found to be a powerful predictor of poor treatment outcomes among HIV positive people in Africa, impaired immune reconstitution, and delayed clinical recovery (54, 55). Low hematocrit and low mean corpuscular hemoglobin concentration (MCHC) were also found to be strongly associated with virological instability (56, 57). These abnormalities are probably a result of chronic inflammation, nutritional deficiencies and impaired bone marrow function. Abnormalities of the white blood cells indicated further evidence of immune system depression. Poor viral suppression was significantly associated with lymphocytopenia, identifying direct destruction of CD4 + T lymphocytes by the uncontrolled HIV replication (58). Those features of long-term viral replication— namely extensive apoptosis of CD4 + cells and generalized immune exhaustion— add to reduced capacity of the host to support viral suppression with ART (59, 60). Poor virological outcomes also were associated with neutropenia and was probably a marker of advanced bone marrow suppression. Neutropenia is also known to make HIV sufferers more vulnerable to severe bacterial infections (61, 62).

### 4.9 Associated factors influencing suboptimal viral suppression

Our logistic regression analysis also revealed that the interaction of structural, social, cultural, and clinical factors influences viral suppression. The odds of suboptimal suppression were slightly lower among women than men, most notably due to better engagement in healthcare. Better treatment outcomes were observed for married participants, which could be linked to the relatively higher level of social support, which in turn helps adherence and fosters sustained engagement in treatment. High pill burden, treatment fatigue, and complexity of therapeutic regimens over time, especially in the context of multimorbidity, are the key barriers to optimal adherence that affect treatment effectiveness (18, 63). It is interesting that the participants who were told about the role of secondary infection showed better viral suppression, probably due to the higher awareness factor influencing participants and encouraging them to act more strictly regarding they adherence. The phenomenon described above had also been referred to as the “cue to action” (2), where acute illness was linked to positive behavioral change, which in turn linked to increased treatment adherence. Geographic accessibility was found to be an important virological determinant. Interestingly, the suppression rates were greater among people who lived further away from treatment centers. As HIV patients choose to avoid competing HIV clinics, they risk unintentionally revealing their status to people around them, thereby causing stigmatization especially in densely populated rural areas (6). As a result, positive patients will attempt to move further apart to safeguard their anonymity and confidentiality, in treatment. Having healthcare facilities readily available had a significant positive impact on virological outcomes, highlighting the need for shorter waiting periods, better health services, and uninterrupted ART supply chains. Healthcare access is also vital to meeting global targets for HIV treatment, as noted in a related report by earlier studies (1, 64). Stigma continued to be a deeply entrenched obstacle to effective viral suppression due to HIV. Meaningful relationship was seen in the virological outcomes of those participants who reported experiencing stigma. Similar studies reported that stigma attached to the use of medications, discourages them from attending drug comprehensive care clinics, and forces patients to hide medications, stop using them, leading to increased risk of virological failure and the development of drug resistance strains (7, 11). Viral suppression was also affected by cultural/religious factors to a negative extent. Some religious and traditional belief systems encourage alternative spiritual healing methods and discourage long-term adherence to ART, as indicated by (4). Patients who drop out of ART to take faith healing or traditional medicine will have the high incidence of virological rebound, rapid progression of the disease.

#### 4.9.1 Limitations of the study

Several limitations were noted in this the study. First of all, the study design is a cross-sectional study, which does not allow definite causal or temporal conclusions to be drawn regarding the relationship between the types of predictors which were identified and viral suppression. Secondly, although tuberculosis (TB) is an important HIV comorbidity whose effect on immune function and treatment response is well established, it was not extensively examined in relation to viral suppression. We again noticed that, even with the achieved sample size within three large ART centers, the sample might not be totally sufficient for detecting associations with rare clinical conditions, opportunistic infections and laboratory abnormalities which could diminish statistical power for some variables. We further realized that, recall or reporting bias could occur with the use of clinical records and self-reports of adherence. In addition to, although there was an investigation of systemic infections and comorbidities these were not associated with the virological outcomes, which may also be due to these sample size limitations. Finally, there was no information on concurrent CD4 cell counts, which prevented assessment of immunological recovery (particularly in regard to people with undetectable viral load), and there was no evidence of any longitudinal follow-up to assess longer-term treatment trajectories, past drug resistance, or overall duration of viral suppression.

## 5 Conclusion

This study highlights the fact that despite ART becoming a golden standard of care for many patients with HIV/AIDS in Upper East Region of Ghana, a large proportion of those on treatment struggle to achieve optimal or suboptimal viral suppression. These findings suggest that clinical and systemic healthcare-related factors contribute to virologic suppression together. In particular, stool, urine, and blood have the potential of containing undetected systemic co-infections, which when not properly handled, pose a huge level of physiological stress to the patient and compromises the effectiveness of ARTs. Moreover, unequal access to medicines, distance to care, prevailing social stigma and cultural factors continue to be important factors when it comes to treatment failure. These findings highlight an extremely strong argument for the need to move toward holistic patient-based management to ensure effective viral control over the long term. This calls for enhanced capacity for routine diagnostics and infection testing at the facility level, tracking down clinical delays, and bolstering community-based support to address structural barriers. Finally, the results of the research presented here suggest that a concerted clinical and public health strategy is required to find the maximum success for ART in limited-resource contexts. Reaching the global goal (95–95-95) of sustained virological suppression is dependent solely on embedding strong clinical surveillance, patient adherence support, and system-wide structural change to simplify health outcomes for people living with HIV(PLWHIV) in the region.

## Patient consent

The participants possessed complete awareness regarding the purpose of the study, procedures, risks it could expose them to plus the expected benefits and were given the opportunity to pose questions prior to the studies. Literate participants were asked to give written consent and participants unable to do so gave their thumbprint consent. The interviews and sample collection took place in privacy to create confidentiality and use coded identifiers to facilitate anonymity. The risk was low and the personal gains were nonexistent. No one was compensated in terms of money or material award and could withdraw any time without repercussions hence this was totally voluntary.

## Data Availability

The raw data supporting the conclusions of this article will be made available by the authors, without undue reservation.

## Glossary

3TC: Lamivudine
AIDS: Acquired immunodeficiency syndrome
AOR: Adjusted Odds Ratio
ART: Antiretroviral Therapy
AZT: Zidovudine
CART: Combination Antiretroviral Therapy
CCR5: C- C Chemokine Receptor Type 5
CD4 +: Cluster of Differentiation 4 Positive T Cells
CI: Confidence Interval
C-X-C: Chemokine Receptor Type 4 (CXCR4-)
DNA: Deoxyribonucleic Acid
HAART: Highly Active Antiretroviral Therapy
H. I. V: Human Immunodeficiency Virus
MS: Metabolic Syndrome
PLWHIV: People Living with HIV
RNA: Ribonucleic Acid
TLD: Tenofovir Lamivudine Dolutamivir
TND: Target Not Detected
UNAIDS: Joint United Nations Programme on HIV/AIDS
WHO: World Health Organization
OIs: Opportunistic infections

## References

[1] Orlando S Silaghi LA Cicala M Lowole MW Massango C Lunghi R . The global response to HIV/AIDS in sub-Saharan Africa: achievements, challenges, and perspectives for the future. Front Public Health. (2025) 13:1665666. doi: 10.3389/fpubh.2025.1665666, 41246083 PMC12615466

[2] Ifedilichukwu NH Joy OS. "Antiviral Targets and known Antivirals (HAART)". In: Ifedilichukwu NH, editor. Antiviral Strategies in the Treatment of Human and Animal Viral Infections. London: IntechOpen (2023)

[3] Hailu G. *HIV Viral Load Suppression Status and Associated Factors among Pregnant Women Receiving Highly Active Antiretroviral Therapy (HAART) in Ethiopia*. Addis Continental Institute of Public Health. (2024). 10.1186/s12985-025-02659-0 PMC11853537 40001227

[4] Agbeko A Owusu R Alhassan Y Letsa T Axame WK Ogum-Alangea D. HIV status disclosure and quality of life of people living with HIV/AIDS in the ho municipality, Ghana. Adv Public Health. (2022) 2022:1–12. doi: 10.1155/2022/6842957

[5] Boakye DS Adjorlolo S. Achieving the UNAIDS 95-95-95 treatment target by 2025 in Ghana: a myth or a reality? Glob Health Action. (2023) 16:2271708. doi: 10.1080/16549716.2023.2271708, 37921654 PMC10627043

[6] Abubakari A Issah H Mutaka MAO Asumah MN. Determinants of virological failure in HIV patients on highly active antiretroviral therapy (HAART): a retrospective cross-sectional study in the upper east region of Ghana. Venereology. (2023) 2:16–29. doi: 10.3390/venereology2010002

[7] Mutaka MAO. *Determinants of VIROLOGICAL Failure in HIV Patients on Highly Active Antiretroviral Therapy (HAART) in the Upper East Region of Ghana, 2022*. (2022).

[8] Mohammadi H Shooraj M Ehteshaminia Y Mahdavi SA. A review on the co-infection of HIV and parasitic diseases. Tabari Biomed Stud Res J. (2022) 4:8775. doi: 10.18502/tbsrj.v4i1.8775

[9] Sileshi B Asmerom H Gemechu K Diba G Mekonnen S Arkew M. Hematological abnormalities and associated factors among adult HIV/AIDS patients on antiretroviral therapy at Hiwot Fana comprehensive specialized hospital: a cross sectional study. Medicine. (2025) 104:e46223. doi: 10.1097/MD.0000000000046223, 41366907 PMC12689065

[10] Portilla-Tamarit J Reus S Portilla I Fuster Ruiz-de-Apodaca MJ Portilla J. Impact of advanced HIV disease on quality of life and mortality in the era of combined antiretroviral treatment. J Clin Med. (2021) 10:716. doi: 10.3390/jcm10040716, 33670229 PMC7916912

[11] Yunusa E. Patterns of Health Seeking Behaviour Among People Living with HIV/AIDS in Selected Hospitals in Benin City, Edo State. Irvine, CA: UAR Universal Publisher (2025).

[12] Obeagu EI Kanu SN. Aplastic anemia in HIV: management considerations in resource-limited settings. Lifeline J Haematol. (2024) 2:1–14.

[13] World Health Organization. Consolidated Guidelines on HIV Prevention, Testing, Treatment, Service Delivery and Monitoring: Recommendations for a public Health Approach. Geneva: World Health Organization (2021). 34370423

[14] Abdulai K Osman Aleboko S Anane I Addae-Mensah I Bawah A-M. Prevalence and determinants of hypertension among people living with HIV in Ghana: a cross-sectional study. PLoS One. (2026) 21:e0342198. doi: 10.1371/journal.pone.0342198, 41678461 PMC12900339

[15] Bowley AL. Elements of Statistics. London: King (1926).

[16] Kharsany ABM Karim QA. HIV infection and AIDS in sub-Saharan Africa: current status, challenges and opportunities. Open AIDS J. (2016) 10:34–48. doi: 10.2174/1874613601610010034, 27347270 PMC4893541

[17] Shisana O Rehle T Simbayi LC Zuma K Jooste S Zungu N . South African national HIV Prevalence, Incidence and Behaviour Survey, 2012. (2014). 10.2989/16085906.2016.1153491 27002359

[18] Sarma P Cassidy R Corlett S Katusiime B. Ageing with HIV: medicine optimisation challenges and support needs for older people living with HIV: a systematic review. Drugs Aging. (2023) 40:179–240. doi: 10.1007/s40266-022-01003-3, 36670321 PMC9857901

[19] Abuogi LL Humphrey JM Mpody C. Achieving UNAIDS 90-90-90 targets for pregnant and postpartum women in sub-Saharan. J Virus Erad. (2018) 4:33–9. doi: 10.1016/S2055-6640(20)30343-5, 30515312 PMC6248851

[20] Owusu LB Ababio C Boahene S Zakaria A-FS Emikpe AO Dwumfour CK . The predictors of unsuppressed viremia among PLHIV: a cross-sectional study in Ghana. BMC Public Health. (2023) 23:1113. doi: 10.1186/s12889-023-16032-9, 37296400 PMC10257285

[21] Pellowski JA Kalichman SC Matthews KA Adler N. A pandemic of the poor: social disadvantage and the US HIV epidemic. Am Psychol. (2013) 68:197–209. doi: 10.1037/a0032694, 23688088 PMC3700367

[22] Deeks SG Lewin SR Havlir DV. The end of AIDS: HIV infection as a chronic disease. Lancet. (2013) 382:1525–33. doi: 10.1016/S0140-6736(13)61809-7, 24152939 PMC4058441

[23] Armoon B Higgs P Fleury M-J Bayat A-H Moghaddam LF Bayani A . Socio-demographic, clinical and service use determinants associated with HIV related stigma among people living with HIV/AIDS: a systematic review and meta-analysis. BMC Health Serv Res. (2021) 21:1004. doi: 10.1186/s12913-021-06980-6, 34551772 PMC8459487

[24] Ncitakalo N Sigwadhi LN Mabaso M Joska J Simbayi L. Exploring HIV status as a mediator in the relationship of psychological distress with socio-demographic and health related factors in South Africa: findings from the 2012 nationally representative population-based household survey. AIDS Res Ther. (2023) 20:6. doi: 10.1186/s12981-022-00498-5, 36747255 PMC9901137

[25] Venter WDF Moorhouse M Sokhela S Fairlie L Mashabane N Masenya M . Dolutegravir plus two different prodrugs of tenofovir to treat HIV. N Engl J Med. (2019) 381:803–15. doi: 10.1056/NEJMoa1902824, 31339677

[26] Gupta RK Gregson J Parkin N Haile-Selassie H Tanuri A Forero LA . HIV-1 drug resistance before initiation or re-initiation of first-line antiretroviral therapy in low-income and middle-income countries: a systematic review and meta-regression analysis. Lancet Infect Dis. (2018) 18:346–55. doi: 10.1016/S1473-3099(17)30702-8, 29198909 PMC5835664

[27] Sarma P. Ageing with HIV: Exploring Older People’s Experiences with Medicines. United Kingdom: University of Kent (2024).

[28] Blanco N Lavoie M-CC Koech E Riedel DJ Ngeno C Adebajo S . Re-engagement into HIV care: a systematic review. AIDS Behav. (2022) 26:132–46. doi: 10.1007/s10461-021-03365-y, 34245395

[29] Chamberlin S Mphande M Phiri K Kalande P Dovel K. How HIV clients find their way Back to the ART Clinic: a qualitative study of disengagement and re-engagement with HIV Care in Malawi. AIDS Behav. (2022) 26:674–85. doi: 10.1007/s10461-021-03427-1, 34403022 PMC8840926

[30] Boakye P Safowaa A. Prevalence and predictors of viral load suppression in adults living with HIV in the western region of Ghana: a cross-sectional study. AIMS Public Health. (2023) 10:469–79. doi: 10.3934/publichealth.2023033, 37304596 PMC10251059

[31] Kyere GA Vechey GA Charles-Unadike VO Tarkang EE. Trends in viral load suppression among HIV patients on antiretroviral therapy (ART) at Asante Mampong municipal hospital, Ghana: 2019–2023. BMC Infect Dis. (2024) 24:1170. doi: 10.1186/s12879-024-10072-1, 39415134 PMC11483983

[32] Paiardini M Müller-Trutwin M. HIV-associated chronic immune activation. Immunol Rev. (2013) 254:78–101. doi: 10.1111/imr.12079, 23772616 PMC3729961

[33] Moretti S Schietroma I Sberna G Maggiorella MT Sernicola L Farcomeni S . HIV-1–host interaction in gut-associated lymphoid tissue (GALT): effects on local environment and comorbidities. Int J Mol Sci. (2023) 24:12193. doi: 10.3390/ijms241512193, 37569570 PMC10418605

[34] Heron SE Elahi S. HIV infection and compromised mucosal immunity: oral manifestations and systemic inflammation. Front Immunol. (2017) 8:241. doi: 10.3389/fimmu.2017.00241, 28326084 PMC5339276

[35] Deku JG Botchway KA Kinanyok S Gedzeah CK Duneeh RV Duedu KO. Intestinal parasitic infection and associated risk factors among HIV-infected patients Seeking healthcare in a rural Hospital in Ghana. J Pathog. (2022) 2022:1–7. doi: 10.1155/2022/5652637, 36046216 PMC9424049

[36] Akalu TY Aynalem YA Shiferaw WS Merkeb Alamneh Y Getnet A Abebaw A . National burden of intestinal parasitic infections and its determinants among people living with HIV/AIDS on anti-retroviral therapy in Ethiopia: a systematic review and meta-analysis. SAGE Open Med. (2022) 10:1082450. doi: 10.1177/20503121221082447, 35284074 PMC8908390

[37] Cumming E Peters C. Immune response to infection. Anaesth Intensive Care Med. (2024) 25:439–43. doi: 10.1016/j.mpaic.2024.04.003

[38] Grinspoon SK. Metabolic syndrome and cardiovascular disease in patients with human immunodeficiency virus. Am J Med Suppl. (2005) 118:23–8. doi: 10.1016/j.amjmed.2005.01.047, 15903292

[39] Rudich A Ben-Romano R Etzion S Bashan N. Cellular mechanisms of insulin resistance, lipodystrophy and atherosclerosis induced by HIV protease inhibitors. Acta Physiol Scand. (2005) 183:75–88. doi: 10.1111/j.1365-201X.2004.01383.x, 15654921

[40] Obirikorang C Quaye L Osei-Yeboah J Odame EA Asare I. Prevalence of metabolic syndrome among HIV-infected patients in Ghana: a cross-sectional study. Niger Med J. (2016) 57:86–90. doi: 10.4103/0300-1652.182082, 27226681 PMC4872497

[41] Iacob SA Iacob DG Jugulete G. Improving the adherence to antiretroviral therapy, a difficult but essential task for a successful HIV treatment—clinical points of view and practical considerations. Front Pharmacol. (2017) 8:831. doi: 10.3389/fphar.2017.00831, 29218008 PMC5703840

[42] Kaspar MB Sterling RK. Mechanisms of liver disease in patients infected with HIV. BMJ Open Gastroenterol. (2017) 4:e000166. doi: 10.1136/bmjgast-2017-000166, 29119002 PMC5663263

[43] Sherman KE Thomas DL. HIV and liver disease: a comprehensive update. Top Antivir Med. (2022) 30:547–58. doi: 10.1080/09540121.2022.2038363, 36375129 PMC9681142

[44] Marchetti G Tincati C Silvestri G. Microbial translocation in the pathogenesis of HIV infection and AIDS. Clin Microbiol Rev. (2013) 26:2–18. doi: 10.1128/CMR.00050-12, 23297256 PMC3553668

[45] Serrano-Villar S Rojo D Martínez-Martínez M Deusch S Vázquez-Castellanos JF Bargiela R . Gut Bacteria metabolism impacts immune recovery in HIV-infected individuals. EBioMedicine. (2016) 8:203–16. doi: 10.1016/j.ebiom.2016.04.033, 27428431 PMC4919658

[46] Tamber SS Bansal P Sharma S Singh RB Sharma R. Biomarkers of liver diseases. Mol Biol Rep. (2023) 50:7815–23. doi: 10.1007/s11033-023-08666-0, 37482588

[47] Hunt PW. HIV and inflammation: mechanisms and consequences. Curr HIV AIDS Rep. (2012) 9:139–47. doi: 10.1007/s11904-012-0118-8, 22528766

[48] Ifeyinwa E Obiechefu MC Adnan M Olayemi OS Deborah D Boluwatife CCU . The imbalance of body electrolytes in HIV patients using highly active antiretroviral therapy. Children (Basel). (2023) 5:1–100.

[49] Obirikorang C Osakunor DNM Ntaadu B Adarkwa OK. Renal function in Ghanaian HIV-infected patients on highly active antiretroviral therapy: a case-control study. PLoS One. (2014) 9:e99469. doi: 10.1371/journal.pone.0099469, 24921259 PMC4055675

[50] Smyth B Haber A Hennessy A. "Kidney Disease and Electrolyte Disorders in the Context of Drug Use". In: Smyth B, editor. Textbook of Addiction Treatment: International Perspectives. Berlin: Springer (2020). p. 1113–32.

[51] Asirvatham ES Ranjan V Garg C Sarman CJ Periasamy M Yeldandi V . A review of Tenofovir Disoproxil fumarate associated nephrotoxicity among people living with HIV: burden, risk factors and solutions. Clin Epidemiol Glob Health. (2024) 25:101462. doi: 10.1016/j.cegh.2023.101462

[52] Wang M Wang Z Chen Y Dong Y. Kidney damage caused by obesity and its feasible treatment drugs. Int J Mol Sci. (2022) 23:747. doi: 10.3390/ijms23020747, 35054932 PMC8775419

[53] Obeagu EI Obeagu GU Alum EU Ugwu OPC. *Anemia as a Prognostic Marker for Disease Progression in HIV Infection*. (2023).

[54] Techane MA Anlay DZ Tesfaye E Agegnehu CD. Incidence and predictors of anemia among children on antiretroviral therapy at the University of Gondar Comprehensive Specialized Hospital, Northwest Ethiopia, 2007–2017: a retrospective follow-up study. HIV AIDS Res Palliat Care. (2020) 12:951–62. doi: 10.2147/HIV.S282675, 33364852 PMC7751604

[55] Sisay Zewdu W Molla Zeleke M Ferede YA Kassie AB Singh P Alemu MA . Unveiling the prevalence of anaemia and its predictors among adults on highly active antiretroviral therapy in the dolutegravir era: a retrospective cross-sectional study. BMJ Open. (2024) 14:e086480. doi: 10.1136/bmjopen-2024-086480, 39242159 PMC11590816

[56] Tilahun M Gedefie A Ebrahim E Seid A Ali A Shibabaw A . Immuno-haematological abnormalities of HIV-infected patients before and after initiation of highly active antiretroviral therapy in the antiretroviral therapy clinics of six health facilities at Dessie town, Northeast Ethiopia. J Blood Med. (2022) 13:243–53. doi: 10.2147/JBM.S364700, 35592587 PMC9112337

[57] Malapati B Nadeem SM Shah MM. Analysis of blood parameters in HIV positive patients. Int J Clin Biochem Res. (2020) 7:388–94. doi: 10.18231/j.ijcbr.2020.083

[58] Obeagu EI. Cytopenias with HIV in the era of highly active antiretroviral therapy: are we winning the Battle? Health Sci Rep. (2026) 9:e72065. doi: 10.1002/hsr2.72065, 42022595 PMC13097676

[59] Westerdijk T. *Decoding HIV Dynamics: The Role of Modeling in Understanding Viral Load Decay under Retroviral Therapy*. (2024).

[60] Okoye AA Duell DD Fukazawa Y Varco-Merth B Marenco A Behrens H . CD8+ T cells fail to limit SIV reactivation following ART withdrawal until after viral amplification. J Clin Invest. (2021) 131:e141677. doi: 10.1172/JCI141677, 33630764 PMC8262469

[61] Moore RD Keruly JC Chaisson RE. Neutropenia and bacterial infection in acquired immunodeficiency syndrome. Arch Intern Med. (1995) 155:1965–70. doi: 10.1001/archinte.1995.00430180067008, 7575050

[62] Levine AM Karim R Mack W Gravink DJ Anastos K Young M . Neutropenia in human immunodeficiency virus infection: data from the women’s interagency HIV study. Arch Intern Med. (2006) 166:405–10. doi: 10.1001/archinte.166.4.405, 16505259

[63] Frey E Johnston CD Siegler EL. Treatment regimens and care models for older patients living with HIV: are we doing enough? HIV AIDS Res PalliatCare. (2023) 15:191–208. doi: 10.2147/HIV.S311613, 37153650 PMC10155713

[64] Hailu G Keraleme A Zealiyas K Tesema A Nuramed N Girmachew F . Human immunodeficiency virus (HIV) viral load suppression status and associated factors among pregnant women receiving highly active antiretroviral therapy (HAART) in Ethiopia. Virol J. (2025) 22:49. doi: 10.1186/s12985-025-02659-0, 40001227 PMC11853537

